# Dedifferentiation of Foetal CNS Stem Cells to Mesendoderm-Like Cells through an EMT Process

**DOI:** 10.1371/journal.pone.0030759

**Published:** 2012-01-20

**Authors:** Suzan Ber, Caroline Lee, Octavian Voiculescu, M. Azim Surani

**Affiliations:** 1 Wellcome Trust/Cancer Research UK Gurdon Institute of Cancer and Developmental Biology, University of Cambridge, Cambridge, United Kingdom; 2 Department of Physiology, Development and Neuroscience, University of Cambridge, Cambridge, United Kingdom; City of Hope National Medical Center and Beckman Research Institute, United States of America

## Abstract

Tissue-specific stem cells are considered to have a limited differentiation potential. Recently, this notion was challenged by reports that showed a broader differentiation potential of neural stem cells, *in vitro* and *in vivo*, although the molecular mechanisms that regulate plasticity of neural stem cells are unknown. Here, we report that neural stem cells derived from mouse embryonic cortex respond to Lif and serum *in vitro* and undergo epithelial to mesenchymal transition (EMT)-mediated dedifferentiation process within 48 h, together with transient upregulation of pluripotency markers and, more notably, upregulation of mesendoderm genes, *Brachyury* (T) and *Sox17*. These induced putative mesendoderm cells were injected into early gastrulating chick embryos, which revealed that they integrated more efficiently into mesoderm and endoderm lineages compared to non-induced cells. We also found that TGFβ and Jak/Stat pathways are necessary but not sufficient for the induction of mesendodermal phenotype in neural stem cells. These results provide insights into the regulation of plasticity of neural stem cells through EMT. Dissecting the regulatory pathways involved in these processes may help to gain control over cell fate decisions.

## Introduction

Major cell fate decisions occur early in embryonic development when all three germ layers are established during gastrulation. This is achieved at the level of the primitive streak, by epithelial to mesenchymal transitions (EMT), where embryonic epiblast cells lose their typical epithelial character with tight cell-cell junctions and acquire a mesenchymal morphology with loose cell-cell contacts and high motility [1], [2], [3], [4]. TGFβ is considered a major inducer of EMT, initiating Smad-mediated genetic programming [5], [6]. However, cross-talk with other signalling pathways like PDGF [7], Wnt [8], [9], Notch [10], [11] and Shh [12], [13] has been shown to be essential for EMT to occur. One hallmark of EMT is the downregulation of epithelial markers like *E-cadherin*s, a process regulated by transcription factor *Snail2 (Slug)* [14], and the upregulation of mesenchymal markers *N-cadherins* [3]. The epithelial and mesenchymal phenotypes are not irreversible. Indeed, during further stages of development cells within the germ layers undergo several rounds of EMT and/or its opposite, mesenchymal to epithelial transition (MET) to form various tissues and organs [4]. Moreover, there is emerging evidence indicating that EMT as well as MET have a pivotal role in regulating cell plasticity and re-establishing tissue integrity, which also occurs during wound healing and regeneration in adults [15], [16].

EMT (as well as MET) is a well orchestrated event involving major cytoskeletal reorganization, cell-cell, cell-ECM interactions and major epigenetic rearrangements leading to changes in expression levels of nearly 4000 genes [17]. A number of recent reports suggest that EMT and MET are also key processes during dedifferentiation and may lead to generation of stem cells [18], [19], [20]. For instance, Li and colleagues [18] have shown that MET is a necessary process during reprogramming cells of mesenchymal origin like fibroblasts to iPS (induced pluripotent stem) cells, and blocking MET impaired the reprogramming process. Other recent studies [20], [21], [22] have shown that *Snail* or *Twist* (transcription factors associated to EMT process) proteins are involved in acquisition of stem cell characteristics in epithelial cancer cells indirectly regulating the expression of pluripotency genes such as *Oct4* and *Nanog*, suggesting an involvement of EMT in dedifferentiation of cells of epithelial origin and establishment of the stem cell signature.

EMT also plays a fundamental role in the emigration of neural crest cells from the neural tube [3], [23] to different tissues, e.g. craniofacial bone, cartilage, smooth muscle, peripheral nervous system [23]. Dorsal neural tube contains pre-migratory neural crest cells that undergo EMT under inductive signals like TGFβ (i.e. BMP4), Wnt, FGF and Notch. Similar signalling cascades operate during EMT at gastrulation and neural crest migration. Migratory neural crest cells downregulate cadherins like E-cadherin and Cadherin 6B [24] and delaminate from neural tube towards different regions of developing embryo where they reaggregate via MET and undergo differentiation (reviewed in [4]). Neural stem cells found in the central nervous system (CNS), on the other hand are direct descendants from the neural tube. Until recently, CNS stem cells have been considered to have a limited differentiation potential. However, it appears that CNS stem cell fate is restricted mostly by the local microenvironment. A previous report suggested that embryonic and adult neural stem cells acquire neural crest phenotype *in vitro* through an EMT process in response to BMPs and FGFs signalling [25]. Furthermore, more recently, adult CNS precursors with restricted differentiation potential, have been shown to generate cell lineages of neural crest lineage during axonal regeneration, indicating the effect of microenvironment on plasticity of CNS derived stem/progenitor cells *in vivo* [26].

With the aim to explore the plasticity of neural stem cells, we exposed mouse embryonic cortex derived neural stem cells to different inductive environment *in vitro* and *in vivo*. Here we show that neural stem cells from brain cortex of E12.5 embryo can dedifferentiate *in vitro* to mesendoderm-like cells and acquire a broader differentiation potential than observed earlier in response to Lif/serum. This dedifferentiation is accompanied by an EMT process and results in the upregulation of pluripotency (e.g., *Nanog*, *Oct4*) and mesendoderm markers (e.g., *Brachyury* and *Sox17*). The JAK/STAT3 and TGFβ pathways are apparently necessary for expression of mesendoderm markers in induced neural stem cells. Furthermore, we show *in vivo* that the induced neural stem cells contribute effectively to both mesoderm and endoderm lineages more efficiently than the non-induced cells when injected into early gastrulating chick embryos.

## Results

### Neural stem cells cultured in serum and Lif display upregulation of pluripotency markers

**Figure 1:**
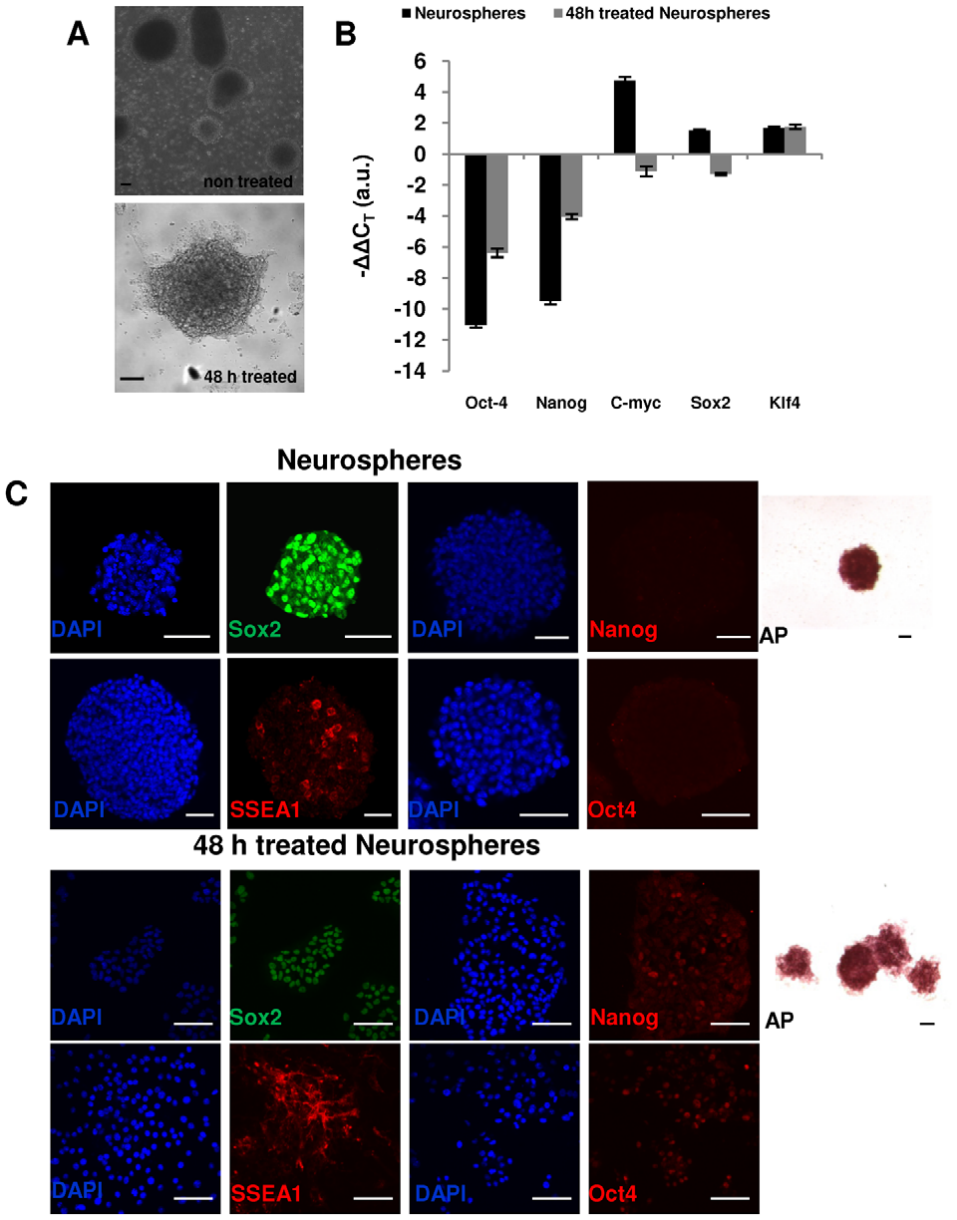
Expression of pluripotency associated proteins in serum-free cultured neurospheres and 48 h serum and Lif induced neurospheres. (**A**): Morphology in serum-free (upper panel) and 48 h serum/Lif (lower panel) conditions. (**B**): Transcriptional profile of pluripotency associated factors Oct4, Sox2, c-Myc, Klf4 and Nanog of serum-free cultured neurospheres and 48 h treated neurospheres. Data is expressed as ΔΔCT and normalized to ES cells. (**C**): Immunostaining for Oct4, Nanog, Sox2, SSEA1 and alkaline phosphatase. Abbreviations: AP, alkaline phosphatase; SSEA1, stage specific embryonic antigen 1. Scale bars: 50 µm.

Neurospheres were derived from mouse embryonic cortex at E12.5 and typically expanded for 5 passages in serum-free N2B27 media supplemented with EGF and bFGF. Dissociated neurospheres were then exposed to serum and Lif to explore the effects on the potency of neural stem cells. This transition to the standard conditions used to support self renewal of mouse embryonic stem cells induced major morphological changes within 48 h. Dissociated neurospheres formed flat adherent colonies (Fig. 1A). The changes in cell morphology were accompanied by upregulation of pluripotency genes as judged by real-time PCR analysis in two independent studies (Fig. 1B). Notably, there were detectable levels of Oct4 and Nanog expression in 48 h treated neurospheres, which were significantly higher compared to untreated neurospheres; however, expression levels still remained relatively low when compared to ES cells (Fig. 1B). While *Klf4* levels did not change significantly upon treatment, both *c-Myc* and *Sox2* levels dropped to about half the expression relative to ES expression levels. At protein levels, 85% of the cells dissociated from untreated neurospheres showed strong Sox2 expression (Figure S1A, Fig. 1C) and high alkaline phosphatase (AP) staining; SSEA1 was detected in a heterogeneous population (around 23%, Figure S1B, Fig. 1C), but *Oct4* and Nanog were undetectable (Fig. 1C). By contrast, after 48 h of treatment, Nanog and Oct4 proteins were detectable in a heterogeneous population while SSEA1 became more homogeneously expressed similarly to Sox2 and AP staining.

### Induction of EMT process and upregulation of mesendoderm markers Brachury and Sox17

**Figure 2:**
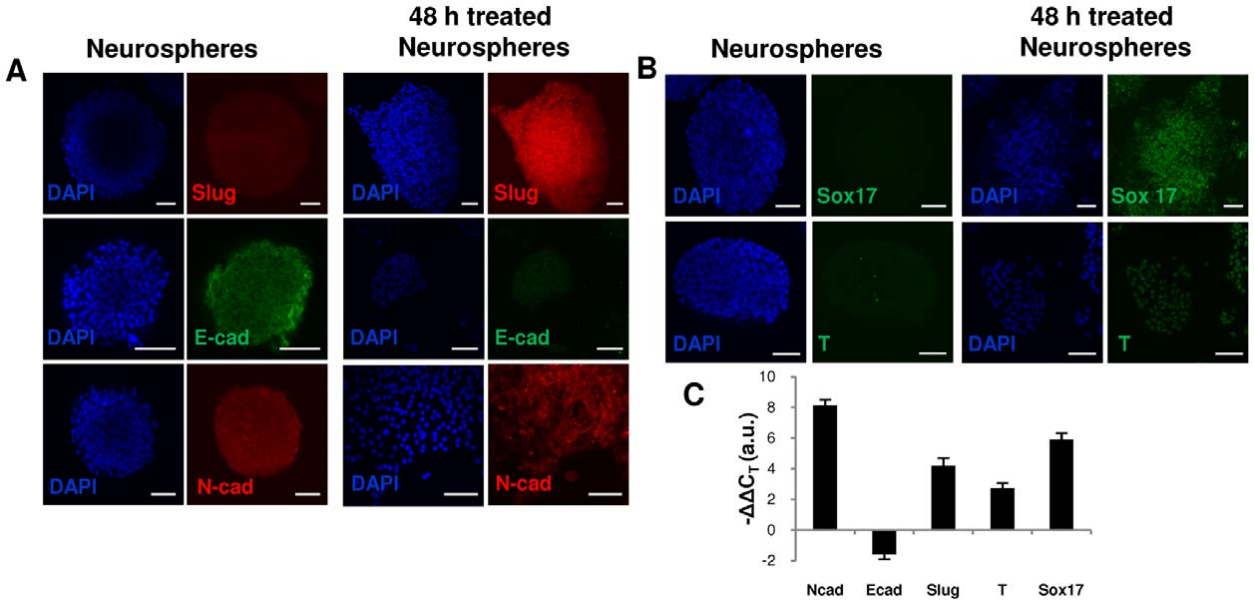
EMT and mesendoderm markers upregulated in neurospheres after 48 h induction with serum and Lif. (**A**): Immunostaining for Slug, E-cadherins and N-cadherins (**B**): Immunostaining for mesendoderm markers Sox17 and Brachyury (T). (**C**): Expression profile of Slug, N-cadherins, E-cadherins, Sox17 and Brachyury of 48 h serum and Lif induced neurospheres measured by QPCR relative to neurospheres cultured in standard neural stem cell media. Abbreviations: Ncad, N-cadherins; Ecad, E-cadherins. Scale bars: 50 µm.

Neural stem cells can be cultured in serum-free conditions as non-adherent floating neurospheres. Neurospheres express high levels of E-cadherins, while N-cadherins are weakly expressed and not localized on the cells surface (Fig. 2A). Following 48 h induction, N-cadherins localized at the cell membrane; and there was a marked change in E- to N- cadherins ratio both in mRNA and protein levels (Fig. 2A, C); this is a hallmark of the EMT process. This change from *E* to *N* cadherin provides motility to the cells. When neurospheres, composed of tightly bound epithelial cells, were left in serum and Lif conditions during the first 48 h those epithelial cells at the edges of the neurospheres acquired flat, spindle-like morphology and start to dissociate and migrate away from the parental epithelial colony (Figure S3). In order to confirm further the occurrence of an EMT, we quantified the mRNA levels of *Slug* (*Snail2*), an essential transcription factor of the EMT process, and other important EMT associated transcription factors *Twist, Goosecoid (Gsc), Sox10*. Consistent with EMT process, *Slug* (Figure 2A, C), *Twist, Gsc* and *Sox10* (Figure S3) showed significant upregulation in 48 h induced neurospheres when compared to serum-free cultured neurospheres.

Interestingly, the expression of pluripotency and EMT markers was accompanied by the expression of mesendoderm markers *Brachyury* and *Sox17* in 48 h induced cells (Figure 2 B, C). These two mesendoderm markers were also expressed homogeneously by all cells with clear nuclear localization consistant with a mesendoderm phenotype [27], [28], [29].

**Figure 3:**
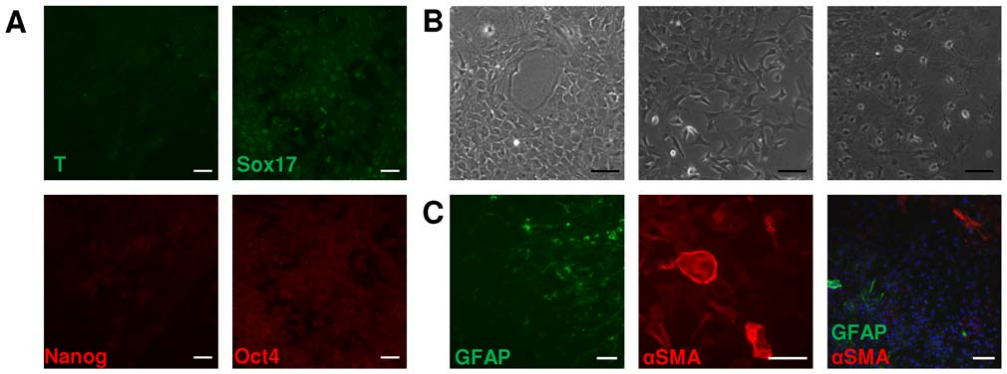
*In vitro* differentiation of neurospheres in serum and Lif conditions. (**A**): Immunostaining for Brachyury (T), Sox17, Nanog and Oct4 at 5 days after induction in serum and Lif conditions (**B**): At 10 days post induction with serum and Lif, cells exhibit very heterogeneous morphologies, indicating the presence of different cell types. (**C**): Immunostaining for GFAP and αSMA in 10 day induced cultures. Abbreviations: GFAP, glial fibrillary acidic protein; αSMA, alpha smooth muscle actin. Scale bars: 50 µm.

The expression of both pluripotency and mesendoderm markers were transient, and decreased significantly after 5 days in culture (Fig. 3A). After 10 days cells exhibited very different morphologies in regions at different densities (Fig. 3B), as they exhibited evidence of differentiation to glial cells (as judged by the expression of GFAP) and to alpha smooth muscle actin (αSMA) positive cells (Fig. 3C); many cells that did not stain for either marker persisted in culture suggesting possible differentiation to other cell types (Fig. 3C, co-staining).

### Fate of cells induced for 48 h in early gastrulating chick embryos

**Figure 4:**
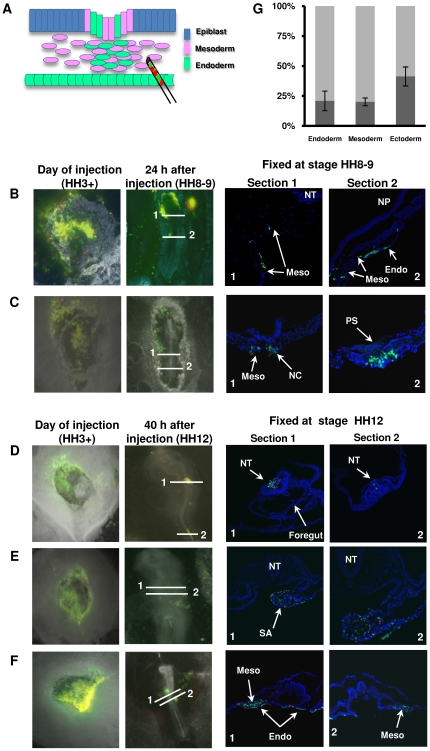
*In vivo* integration potential of 48 h serum and Lif induced neurospheres. (A): Diagram showing injection site of induced (green) and non-induced (red) neurospheres in HH3+ chick embryos. B,D show images taken immediately after injection, and 24 h (stage HH8-9) post injection of two different embryos. Cross section images correspond to planes indicated on representative images of fixed embryos 24 h after injection. (**B**): Cross sections show successful integration of injected cells in the lateral plate mesoderm and endoderm (1, 2). (**C**): both green and red labelled cells are detected in notochord and somite mesoderm (1). However, primarily green labelled cells have been detected ingressing from the late primitive streak (2). D, E and F show images taken immediately after injection and 40 h (stage HH12) post-injection of three different embryos. Cross section images correspond to planes indicated on representative images of fixed embryos 40 h after injection. (**D**): Cross section images showing injected cells within the neural tube (1) and hindbrain region (2). (**E**): Cross section images showing the incorporation of injected cells into sinoatrial region of the embryo (1, 2). (**F**): Cross sections show successful integration of injected cells in endoderm as well as lateral plate mesoderm (1, 2). (**G**): The chart shows proportion of green (48 h induced) and red (non-induced) cells in endoderm, mesoderm and ectoderm layers. 48 h induced cells (grey part) represent around 80% of labelled cells which integrated to mesoderm and ectoderm layers, and around 55% of labelled cells present in the ectoderm. Abreviations: HH stage, Hamburger and Hamilton stage; NC, notochord; NT, neural tube; PS, primitive streak; NP, neural plate; Endo, endoderm; Meso, mesoderm; SA, sinoatrial region.

**Figure 5:**
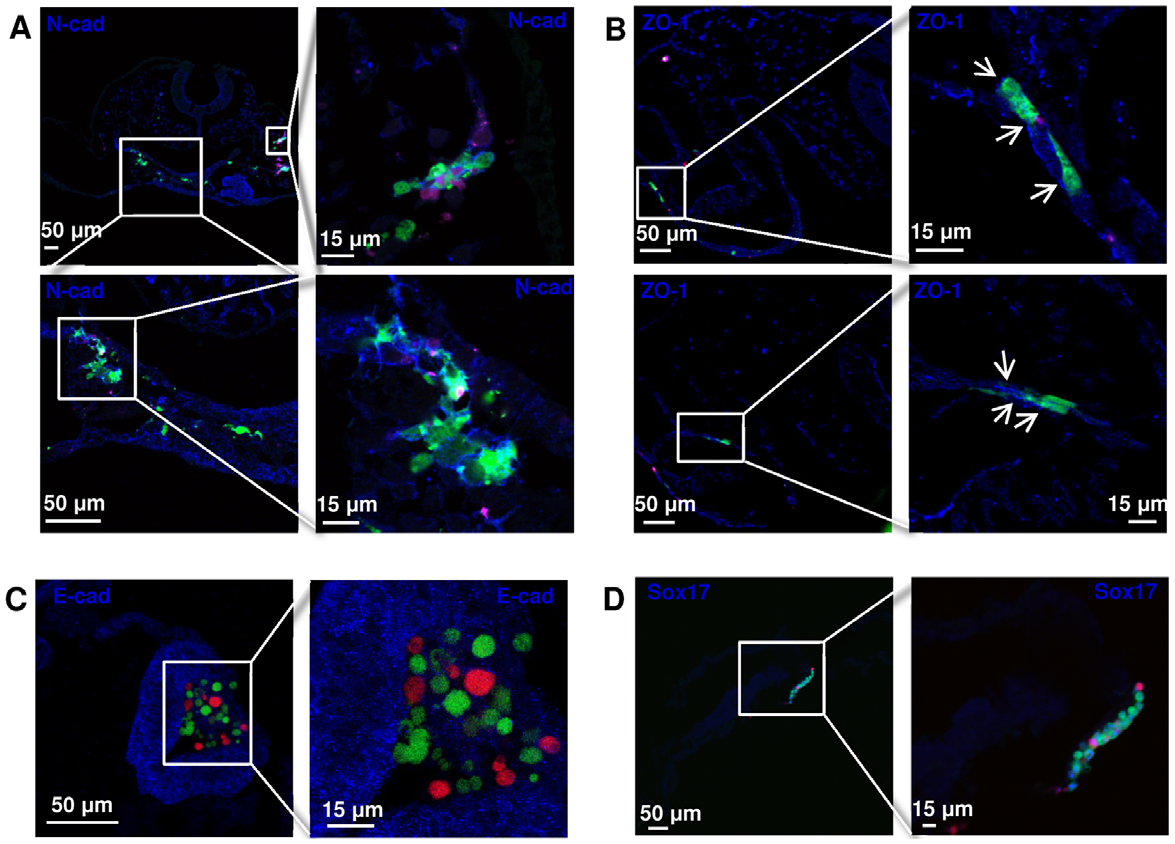
*In vivo* differentiation of 48 h induced neural stem cells. Staining of chick embryo paraffin sections for mesenchymal marker N-cadherin, tight junction marker ZO-1, endoderm marker Sox17, and epithelial marker E-cadherin. Although some of the labelled cells express E-cadherin, the staining pattern suggest the cells do not achieve a complete integration to ectoderm lineage. However, high integration towards mesoderm and endoderm lineages is confirmed by N-cadherin, ZO-1 and Sox17 stainings. Although induced (green) cells show higher efficiency of integration, both induced (green) and non-induced (red) cells once incorporated into these tissues express respective lineage markers and acquire similar morphological characteristics to their neighbouring host cells.

To investigate the *in vivo* potential of the cells with mesendoderm-like phenotype, we introduced them into early gastrulating chick embryos, a proper developmental time point for mesendoderm differentiation, and we tested their ability to contribute also to lineages other than their origin (i.e., endoderm and mesoderm). Non-treated and 48 h treated neurospheres were labelled with red- and green-fluorescent cell tracker dyes, respectively, mixed in equal numbers and injected into early gastrulating chick embryos at stage HH3+ (mid-primitive streak) between the endoderm and mesoderm layers (see sketch in Fig. 4A). This allows a direct comparison of their potential to integrate and contribute to the germ layers of the embryo. After 24 h (Fig. 4 B, C) and 40 h (Fig. 4 D, E, F) of injection, embryos were fixed, and cross sections were obtained from indicated regions of embryos. Induced and non-induced cells were detected at similar ratios in ectodermal tissue (p<0.05) (Fig. 4D,G and Fig. 5C), although injected cells had greater tendency to populate tissues other than their origin, i.e., mesoderm and endoderm. However, the frequency of contribution of treated and non-treated cells into lineages different from their origin was significantly different (p<0.001). In fact, around 80% of the labelled cells that integrated into mesoderm and endoderm were green labelled, serum/Lif treated cells (Fig. 4 G, Fig. 5). Moreover, only induced cells could be seen to mingle with cells delaminating from the epiblast layer in the late primitive streak at stage HH9 (green, Fig. 4C (2)). More anteriorly, both induced (green) and non-induced (red) cells can be seen incorporated into somites and endoderm, but with a much greater propensity of the former.

In order to address whether the cells integrate into different tissues successfully and establish contact with the host cells, we stained for mesenchymal surface marker N-cadherin, tight junction protein zona occludens 1 (ZO-1), neuroepithelial marker E-cadherin and endoderm marker Sox17 (Fig. 5). As shown in Fig. 4G, treated cells exhibit little advantage compared to untreated cells in populating the neural tube. Although some of the labelled cells expressed E-cadherin on the cell surface, the staining pattern and their morphology suggests that treated and untreated cells do not achieve a complete integration (Fig. 5C). The majority of the labelled (particularly EMT induced) cells tend to incorporate more efficiently to tissues of mesendoderm origin (Fig. 5A, B, D). In mesoderm tissue of chick embryos, labelled cells expressed N-cadherin and shared a stellate morphology similarly to the neighbouring host cells. Green labelled cells located in the endoderm acquired a flattened morphology, characteristic of the host tissue, expressing also ZO-1 which is a marker for tight junctions expressed strongly in cells of endodermal origin and also in tightly packed neuroepithelial cells (Fig. 5B). Moreover, serum/Lif induced cells and a few untreated cells detected within endoderm tissue expressed also Sox17 (Fig. 5D) confirming further their integration into endoderm tissue. Collectively, experimental data shows that EMT-induced neural stem cells can integrate effectively to mesendoderm lineages despite their ectodermal origin.

### Jak/Stat and TGFβ pathways are essential for induction of the mesendoderm phenotypes

We next investigated which of the factors present in serum are mainly involved in the transformation by 48 h treatment of neurospheres. It has been suggested that TGFβ pathway is one of the main pathways involved in the EMT process, while cross-talk with other pathways is also crucial [3], [9]. Indeed, bFGF was shown to be necessary for BMP mediated induction of EMT and neural crest cell phenotype in cortex derived neural stem cells [25]. Therefore we investigated here TGFβ, FGF and Lif pathways.

We cultured neurospheres in serum-free N2B27 media supplemented with high concentrations of Bmp4, Activin, Lif and bFGF individually and in different combinations (Supporting Information Table S1). Individual applications of the growth factors did not induce the attachment of dissociated neurospheres, while some combinations led to a better attachment (e.g., Bmp4 with any pair of Activin/Lif/bFGF). None of the growth factors used in this study could induce the upregulation of Brachyury and Sox17 as efficiently as Serum + Lif condition. However, in samples containing TGFβ-related ligand (both BMP4 and Activin) and Lif as well as TGFβ ligands and bFGF Brachyury and Sox17 were upregulated, although not exclusively localised in the nucleus (see Supporting Information Table S1 Figure S2).

**Figure 6:**
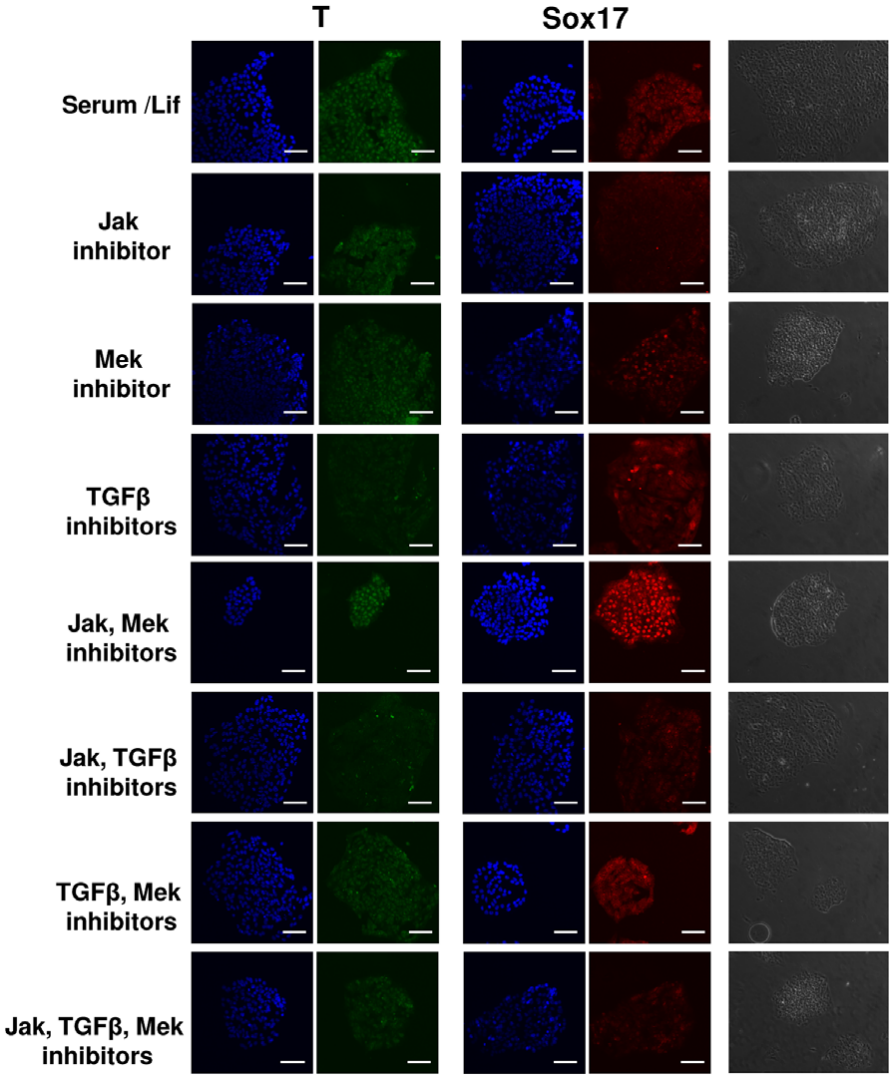
Effect of inhibitors on the expression of mesendoderm markers and morphology of neurospheres. Jak I Inhibitor, Mek inhibitor (PD0325901), TGFβ inhibitors (Noggin+ SB431542) have been used alone or in combination as indicated. The combination of Jak I and TGFβ inhibitors resulted in the most striking inhibition of Brachyury (T) and Sox17 upregulation in neural stem cells. T (green), Sox17 (red). See result section for details. Scale bars: 50 µm.

In a series of complementary experiments, we inhibited each of these pathways, individually or in combinations using inhibitors against Lif (Jak 1 inhibitor), TGFβ (Noggin + SB431542) and FGF (MEK inhibitor PD0325901) pathways. Whereas there were phenotypic changes similar to those observed with Lif/serum, immunostainings exhibited differences in *Brachyury* and *Sox17* expression by the end of the 48 h of treatment. Results summarized in Figure 6 and Table 1 shows that inhibition of individual signalling pathways was not sufficient to abolish completely the mesendoderm phenotype. Mek inhibitor alone did not show any significant effect on Brachyury expression while Sox17 appears upregulated in a modest and heterogeneous manner. Jak1 inhibitor interfered only slightly with Brachyury expression but suppressed Sox17 upregulation; TGFβ inhibitors alone decreased both Brachyury and Sox17 induction also disrupting the nuclear localization of Sox17. Jak1 and TGFβ inhibitors were able to abrogate the expression of Brachury and nuclear Sox17 when applied together. Interestingly, Mek inhibitor opposed the negative regulation of individually applied TGFβ and Jak1 inhibitors on Brachury and Sox17. TGFβ, Jak1 and Mek inhibitors combined however, resulted in much reduced expression levels of Brachyury and Sox17 than the former with disrupted Sox17 nuclear localization. We noted a more striking effect of Mek inhibitor when combined with Jak1 inhibitor which resulted in the increased expression of Sox17 even compared to the serum/Lif conditions.

**Table 1:** Effect of signalling pathway inhibitors on upregulation of mesendoderm markers during 48 h induction of neurospheres.

Samples			Morphology	Brachyury(Nucleus/Cytoplasm)	Sox 17(Nucleus/Cytoplasm)
Serum+Lif			Flat	+++/−	+++/+
**Inhibitors**					
Jak	Mek	TGFβ			
+	−	−	Flat	++/+	−/−
−	+	−	Flat	+++/−	+++/+
−	−	+	Flat	+/+	+/+
+	+	−	Flat	+++/−	++++/+
+	−	+	Flat	−/−	−/+
−	+	+	Flat	++/−	++/++
+	+	+	Flat	+/+	−/+

Expression levels: ++++ very strong; +++ strong; ++good; + weak; − no expression.

We can conclude that the inhibition of both TGFβ and Jak/Stat pathways strongly synergized to inhibit the upregulation of Brachyury and Sox17. Taken together, the results suggest that TGFβ and Jak/Stat pathways are necessary for neurospheres to acquire a mesendoderm-like phenotype (Fig 6, Table 1), but not sufficient to complete this transformation (Supporting Information Figure S2, Table S1).

## Discussion

The long accepted concept of limited differentiation potential of adult stem cells have been challenged by many studies reporting the extended potential of adult stem cells to lineages distinct from their origin [26], [30], [31], [32], [33], [34]. This might be because stem cells may not fully exhibit their full differentiation potential in their confined microenvironment, which becomes evident in an inductive environment *in vitro*. Since neural stem cells already show high expression of *Sox2*, *c-Myc*, *Klf4*, and only need exogenous *Oct4* to be reprogrammed to iPS [35], they may be amenable to dedifferentiation towards, for instance, mesendoderm phenotype.

**Figure 7:**
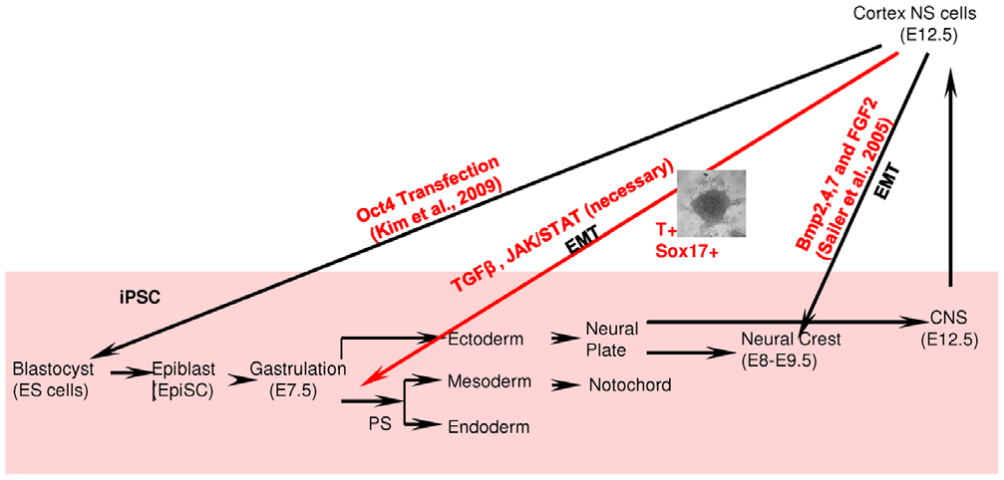
Schematic diagram depicting our interpretation of the results. Neural stem cells derived from later stages of development have been shown to reprogram to iPS cells via overexpression of *Oct4* transcription factor (Kim et al., 2009). Signalling alone (BMPs and bFGF), however, can induce dedifferentiation of neural stem cells into a neural crest phenotype (Sailer et al., 2005). TGFβ and Jak/Stat pathways can induce a further dedifferentiation to mesendoderm-like phenotype providing evidence for extracellular signaling regulated cell plasticity of neural stem cells (this work, red arrow).

Indeed, the key finding of our work is the upregulation of mesendoderm markers *Brachyury* (T) and *Sox17* in neural stem cells associated with an EMT transition after 48 h of serum and Lif treatment. Sailer and colleagues [25] have previously shown that neural stem cells can acquire neural crest cell properties *in vitro* entering an EMT transition when treated with BMP2 (or BMP4) and bFGF. In addition to the expression of EMT-specific markers (i.e., *N-cadherins*, *Slug, Twist, Gsc, Sox10*), here we show *Brachyury* and *Sox17* upregulation in serum and Lif induced neural stem cells to indicate dedifferentiaton beyond neural crest stage. Figure 7 shows a diagrammatic representation of our interpretation of these results.

Further evidence in support of this interpretation was obtained with an *in vivo* cell fate assay using chick embryo. When injected into early gastrulating chick embryos 48 h induced cells integrated into mesoderm and endoderm lineages more efficiently than non-induced neuropsheres, while both cell types were found in similar proportions in the ectoderm tissue. Injection experiments further show that injected cells tend to populate and integrate more significantly to mesendoderm tissues with respect to their tissue of origin. Consistent with these findings, Bernemann and colleagues [36], have shown that in epiblast cells a mesendodermal phenotype, i.e. expression of Brachyury, correlates negatively with the ability to undergo neuronal differentiation and reprogramming to pluripotent embryonic stem cell state; this suggests that those epiblast cells exhibiting mesendodermal phenotype are “primed” to commit towards mesendoderm lineages. Since distinction between neuroectoderm and mesendoderm occurs in a very controlled way during very early embryonic stages [37], [38], [39] once introduced into embryonic environment those mesendoderm-like cells would follow their predetermined fate of differentiation.

Since we have demonstrated fast and transient signalling-mediated induction to a mesendoderm-like state *in vitro*, a contribution of non-induced neurospheres to lineages other than their origin *in vivo* is not surprising. Therefore, it is quite likely that injected non-induced cells do also respond to the early local inductive developmental signals and initiate similar changes induced by *in vitro* serum and Lif conditions. A previous study suggested that various differentiation signals during developmental stages of an embryo can expand the differentiation potential of neural stem (NS) cells [40], although the degree of plasticity of NS cells which they report is still debated [40]. Here we further show that pretreatment of NS cells *in vitro* with serum and Lif renders neurospheres competent to contributing to mesoderm and endoderm lineages much more effectively. Indeed, the 48 h induced cells integrated in diverse tissue with markedly higher efficiency than non-induced cells. Strikingly, only 48 h treated cells were found delaminating from the late primitive streak of HH9 chick embryos, suggesting that after 48 h induction neural stem cells can acquire a state akin to that of cells much earlier in development.

We have reported evidences that this transient dedifferentiation process is under the control of Jak/Stat ad TGFβ pathways, but crosstalk with other signalling pathways may be crucial. Indeed, the aforementioned differences between our work and Sailer et al. are likely to occur because of the complex interactions between various signalling pathways that are affected by the different culturing conditions. For instance, downstream to TGFβ, Smads can directly induce *Brachyury* [41] and *Sox17* expression [27], [29], [42], however to supress *Brachyury* and *Sox17* upregulation we had to treat cells with Jak1 and TGFβ inhibitors together suggesting a synergistic effect between Stat3 and Smad signalling pathways into the transient transformation process of neurospheres into mesendoderm-like cells. From our growth factor experiments bFGF seemed to cooperate with TGFβ and Lif during upregulation of Brachyury and Sox17 (Figure S2), while inhibitor experiments showed that Mek inhibition lead to enhanced Sox17 expression. MAPK pathway was previously demonstrated to attenuate the expression of Sox17 in zebrafish [43]. Although FGFs are known to participate in early mesoderm and endoderm differentiation, their exact role is not yet clarified. Taken together, assays carried out with inhibitors (Fig. 6 and Table 1) and growth factors (Supporting Information Table S1 and Figure S2) indicate that Jak/Stat and TGFβ signalling pathways are strictly necessary, although not sufficient, for the transient transformation of NS into mesendoderm-like cells.

The functional significance of the transient upregulation of pluripotency markers *Nanog* and *Oct4* that we reported remains unknown. We note that, however, during early differentiation of human ES and mouse epiblast stem cells to mesendoderm lineage *Nanog* was shown to cooperate with Smads to directly induce *Brachyury* expression and to upregulate *Sox17* via direct induction of the *Sox17* regulator *Eomes,* indicating that pluripotency factors are not only involved in maintenance of pluripotency but in a crosstalk with other signalling pathways they can also play crucial role during initial steps of cell differentiation [44].

To summarize, we have shown that under serum and Lif conditions neural stem cells initiate an EMT associated transient dedifferentiation process resulting in the induction of mesendoderm markers *Brachyury* and *Sox17* mediated by Jak/Stat3 and TGFβ signalling pathways. *In vivo* studies in chick embryos showed that these cells with mesendoderm-like phenotype can successfully incorporate into lineages other than their origin demonstrating the high plasticity and broader differentiation potential of neural stem cells.

EMT and MET are transient events associated with cell plasticity where extensive genomic and epigenomic changes take place. Recently Li and colleagues [18] demonstrated that iPS generated from mouse fibroblast (i.e., cells of mesenchymal origin) pass through an MET process that is necessary for the efficient induced reprogramming. It still remains to be elucidated whether a full or partial EMT process would play a similar role in induction of reprogramming of cells of an epithelial origin. EMT inducers are silent during adulthood however EMT and MET can be activated during regeneration processes, including wound healing, kidney, liver and heart regeneration [15], [16], [45], [46]. Abnormal activation of EMT in adults can be detrimental [4]. Accumulating evidence shows association of EMT also with cancer and not just at metastatic stage [20], [47], [48]. Mani et al., have shown that EMT stimulates cancer cells to adopt characteristics of stem cells [20]. Interestingly, EMT-related Snail and Slug transcription factors have been shown to induce a self-renewal program in ovarian cancer by upregulation of *Nanog, Oct4, HDAC1&3 and Bmi1* [22]. More recently, Holmberg and colleagues showed that high grade gliomas coexpress pluripotency transcription factors Oct4, Sox2, Nanog and Klf4 together with mesodermal Brachyury and endodermal Sox17 transcription factors [48]. In light of these studies and our own findings, it is tempting to speculate that dedifferentiation program of neural stem cells which results in activation of stem cell regulatory and early developmental pathways could be crucial mechanism involved also in pathology.

EMT is closely associated with cancer progression. Ansieau and colleagues have recently shown that EMT-associated transcription factors Twist-1 and -2, which are highly expressed in many cancers, override oncogene induced senescence (a critical tumor preventive mechanism) by cooperating with activated Ras to inhibit both p53 and Rb tumor suppressor pathways [49]. Also Slug, another EMT-associated transcription factor, is upregulated in various tumors (including gliomas) and can lead to downregulation of p53 activity [50], [51]. On the other hand, tumor suppressor p53 have been reported to suppress EMT and stem cell related genes by upregulating miRNAs (like *miR-200c*) [52]. Since many tumors exhibit deficiency in p53 activity this may lead also to unsufficient suppression of EMT eventually correlating with poor prognosis [50], [53], [54], [55].

Therefore better understanding of this mechanism and the effect of both extrinsic and intrinsic pathways leading to it may provide opportunities to regulate cell fate decisions, help in more efficient cell fate control and in the development of new therapeutic strategies.

## Materials and Methods

### Isolation, culture and 48 h induction of neural stem cells

Cortices from E12.5 MF1 mice were mechanically dissociated in Hanks' Solution (Invitrogen), plated (4×10^4^ cells/cm^2^) and cultured as non adherent neurospheres in Euromed N (EuroClone)) media supplemented with N2 (Invitrogen), B27 without Vitamin A (Invitrogen), bFGF 20 ng/mL (Invitrogen) and EGF 20 ng/mL (Invitrogen). Neurospheres were expanded for several passages.

For 48 h induction in serum conditions, mechanically dissociated neurospheres were cultured in DMEM/F12 media (Gibco) supplemented with 20% FCS (Gibco) and 1000 U/mL LIF for 48 hours. Jak I inhibitor 0.6 µM (Calbiochem) was used to block LIF-Stat3 pathway; SB431542 10 µM (Tocris Bioscience) and rhNoggin 500 ng/mL (R&D Systems) were used to block the TGFβ pathway; 2 µM PD0325901 (Stemgent) was used to block Mek/Erk pathway. The inhibitors have been used alone or in combinations as indicated in Table 1 and Fig. 6.

For 48 h induction using serum-free conditions with growth factors, dissociated neurospheres were cultured with recombinant human (rh) BMP4 250 ng/mL (R&D Systems), rh Activin A 100 ng/mL (Peprotech) and rh bFGF 100 ng/mL (Invitrogen) individually as well as in combinations as indicated in Supporting Information Fig S1 and Table S1.

### Immunohistochemistry

Cultures were fixed for 10 min in 4% paraformaldehyde (PFA) at room temperature. Cells were permeabilized for 15 min at room temperature in PBS containing 1% BSA and 0.1% Triton-X. Primary antibodies were applied in the same permeabilization solution overnight at 4°C. After washing three times with PBS samples were incubated with secondary antibodies diluted in PBS containing 1%BSA and 0.1% Triton-X at 4°C overnight. Slides were mounted with Vectashield with DAPI (Vector Laboratories) and imaged using Olympus FluoView FV1000 Confocal Microscope. Primary antibodies used were: mouse monoclonal Sox2 (1∶200) (Abcam), rabbit polyclonal Oct4 (1∶100) (Santa Cruz Biotechnology), TG1 (anti-SSEA1 monoclonal antibody, 1∶1, kindly provided by Dr Beverly), rabbit polyclonal Nanog (1∶500) (Cosmo Bio Co., Ltd.), goat polyclonal Brachyury (N-19) (1∶100) (Santa Cruz Biotechnology), goat polyclonal Sox17 (1∶200) (R&D Systems), rabbit polyclonal Slug (1∶200) (Cell Signaling Technology), rat monoclonal E-cadherins (1∶40) (Takara Bio Inc.), and rabbit polyclonal N-cadherins (1∶300) (Abcam), rabbit polyclonal ZO-1 (1∶100) (Invitrogen). All secondary antibodies used were Alexa Fluor dye conjugated (Molecular Probes).

### Alkaline phosphatase (AP) staining

Cells were fixed in 4% PFA for 10 min at room temperature and stained using the AP staining kit (Roche Applied Science) according to manufacturers' instructions, overnight at room temperature in the dark.

### Real-time PCR

Total RNA was isolated using TRIzol (Invitrogen) according to manufacturer's protocol followed by DNase treatment and RNA purification. RNA (1 µg) was reverse transcribed using Superscript III reverse transcriptase (Invitrogen) according to manufacturer's instructions. Quantitative PCR was performed in duplicates in 20 µL reaction mix containing 1X SYBR Green PCR Master Mix and 0.5 µM of each primer. Reaction conditions were as following: 94°C for 10 min, followed by 40 cycles at 94°C for 30 s, 60°C for 30 s, and 72°C for 1 min. GAPDH was used as an internal control. Error bars in all QPCR graphs represent standard deviation from two independent experiments. QPCR primers used can be found in Supporting Information Table S2.

### Labelling and injection of cells into chick embryos

48 hour serum and Lif induced Neurospheres were labelled with green cell tracker dye CMFDA 20 µM (Invitrogen) and non-induced neurospheres were labelled with red cell tracker dye CMTPX 20 µM (Invitrogen) prior to injection. Equal amounts of green and red labelled cells were mixed immediately before injections into gastrulation stage embryos (stage HH3+.[56]). Cells were injected between the ectoderm and endoderm using a fine capillary tube attached to a mouth tube.

Primitive streak stage embryos (HH3+) were cultured in vitro using New culture method [57] as modified in [58], [59]. The cultures were incubated in a humidified box at 38°C for 24–40 h.

### Paraffin sections

After incubation embryos were carefully removed from the membrane and fixed in 4% paraformaldehyde overnight at 4°C. After washing 3 times (for 1 h each) with PBS, embryos were dehydrated sequentially in 50% Ethanol for 1 h, 70% Ethanol for 1 h, 100% Ethanol for 1 h. Embryos were rinsed with Xylene, incubated in 100% Xylene for 1 h, 50% Xylene and 50% paraffin for 1 h and left in paraffin for overnight at 65°C. The embryos were then embedded in paraffin and 8 µm-thin microtome sections were cut. Slides were dried overnight at 37°C, paraffin was removed by treating with Xylene for 5 min and rehydrated sequentially into 100% Ethanol for 2 min, 90% Ethanol for 2 min, 70% Ethanol for 2 min and H_2_O for 5 min. Prior to imaging, slides were mounted with Vectashield with DAPI (Vector Laboratories). Slides which were used for immunostainings were first subjected to antigen retrieval by boiling the deparaffinised slides in Citrate buffer (10 mM, pH 6.0) for 10 min. Slides were imaged using Olympus FluoView FV1000 Confocal Microscope. Spectral bleedthrough of the red and green cell tracers was corrected by linear unmixing with bespoke software developed in MATLAB courtesy of Dr. A. Esposito, MRC Cancer Cell Unit, Cambridge (UK).

Quantification of cells integration shown in Fig. 4G was performed on a total of 350representative sections containing fluorescently labelled cells selected from 15 embryos injected in three independent experiments. Only labelled cells that incorporated in the embryo proper have been counted manually. In total 786 red labelled and 2783 green labelled cells were counted in endoderm tissue (p<0.001); 1619 red and 6977 green labelled cells in mesoderm (p<0.001); 216 red labelled and 276 green labelled cells in the ectoderm (p<0.05). Indicated error bars show standard deviations across different sections.

## Supporting Information

Figure S1 **Flow cytometry analysis of non-treated neurospheres showing around 85% of cells expressing Sox2 and around 23% of cells expressing SSEA1.**
(TIFF)

Figure S2 **Representative images showing the effect of growth factors BMP4, Activin, bFGF and Lif on the expression of mesendoderm markers in neurospheres during the 48 h induction in serum-free N2B27 media.** BMP4, Activin, bFGF and Lif were used alone or in combinations as indicated. Images are representative of combinations of growth factors which supported the attachment of neurospheres. Although not exclusively nuclear, both T and Sox17 were detected in samples containing TGFβ-related ligand (both BMP4 and Activin) and Lif. Also bFGF in combination with TGFβ family seems to upregulate these genes. Abbreviations: B: BMP4; A: Activin; F: bFGF; L: Lif. Scale bar 50 µm.
(TIFF)

Figure S3 **Morphology of neurospheres cultured in serum-free and 48 h serum/Lif conditions.** **(A)**. In serum-free media neuronal stem cells are tightly attached forming floating spheres, while upon induction in serum and Lif, cells start to change morphology, acquire a flat and spindle-like morphology and detach from the main colony. Expression levels for other EMT related transcription factors Goosecoid, Twist and Sox10 shows upregulation of these markers upon serum/Lif induction **(B)**. Data is expressed as ΔΔCT and normalized to Neurospheres in serum-free media.
(TIFF)

Table S1 Effect of signalling molecules on upregulation of mesendoderm markers T and Sox17 in neurospheres at 48 h induction in serum free media**.**
(DOC)

Table S2 Primers used for real time PCR.
(DOC)
