# Supplementary material for: Dedifferentiation of Foetal CNS Stem Cells to Mesendoderm-Like Cells through an EMT Process
Source: PLoS One. 2012 Jan 20;7(1):e30759. doi: 10.1371/journal.pone.0030759 (PMC3262838; doi:10.1371/journal.pone.0030759)
Supplement: Table S1 — Effect of signalling molecules on upregulation of mesendoderm markers T and Sox17 in neurospheres at 48 h induction in serum free media. (DOC) [file pone.0030759.s004.doc]

Table S1

| **Samples** | | |  | **Attachment** | **T (N/C)** | **Sox 17 (N/C)** |
| --- | --- | --- | --- | --- | --- | --- |
| **Serum+Lif** | | |  | **Good** | **+++/-** | **+++/+** |
| **Serum-free media+ Growth Factors** | | |  |  |  |  |
| **Bmp4** | **Activin** | **bFGF** | **Lif** |  |  |  |
| **+** | **-** | **-** | **-** | **No** | **ND** | **ND** |
| **-** | **+** | **-** | **-** | **No** | **ND** | **ND** |
| **-** | **-** | **+** | **-** | **No** | **ND** | **ND** |
| **-** | **-** | **-** | **+** | **No** | **ND** | **ND** |
| **+** | **+** | **-** | **-** | **No** | **ND** | **ND** |
| **+** | **-** | **+** | **-** | **Poor** | **-/-** | **-/+** |
| **+** | **-** | **-** | **+** | **Poor** | **-/-** | **-/+** |
| **-** | **+** | **+** | **-** | **Poor** | **-/++** | **+/++** |
| **-** | **+** | **-** | **+** | **Poor** | **-/+** | **+/+** |
| **-** | **-** | **+** | **+** | **Medium** | **-/++** | **+/+** |
| **+** | **+** | **+** | **-** | **Good** | **-/+** | **++/++** |
| **+** | **+** | **-** | **+** | **Good** | **-/++** | **++/++** |
| **+** | **-** | **+** | **+** | **Good** | **-/++** | **++/++** |
| **-** | **+** | **+** | **+** | **Medium** | **+/++** | **+/++** |
| **+** | **+** | **+** | **+** | **Good** | **+/++** | **++/++** |

**Abbreviations: N/C: nuclear/cytoplasmic; ND: not detected**

**Expression levels: ++++ very strong; +++ strong; ++good; + weak; - no expression.**
