# Supplementary material for: Dedifferentiation of Foetal CNS Stem Cells to Mesendoderm-Like Cells through an EMT Process
Source: PLoS One. 2012 Jan 20;7(1):e30759. doi: 10.1371/journal.pone.0030759 (PMC3262838; doi:10.1371/journal.pone.0030759)
Supplement: Table S2 — Primers used for real time PCR. (DOC) [file pone.0030759.s005.doc]

**Table S**2

| **Gene** | **Forward Primer** | **Reverse Primer** |
| --- | --- | --- |
| Pou5f1 (Oct4) | 5’-CACGAGTGGAAAGCAACTCA | 5’-TTCATGTCCTGGGACTCCTC |
| Nanog | 5’-TACCTCAGCCTCCAGCAGAT | 5’-GCTTGCACTTCATCCTTTGG |
| Sox2 | 5’-GGGTTCTTGCTGGGTTTTGA | 5’-CCTTCCTTGTTTGTAACGGTC |
| C-Myc | 5’-GTGCTGCATGAGGAGACACC | 5’-GCCTCTTCTCCACAGACACC |
| Klf4 | 5’-GGAGAAGACACTGCGTCCAG | 5’-GGGAAGTCGCTTCATGTGAG |
| Cdh1 (E-cadherin) | 5’-CCCTGTCTCTGCAAACCAAAA | 5’-TGCTTCCTGAGAAAATGCACAA |
| Cdh2 (N-cadherin) | 5’-GTCATCACGGTGACAGATGTC | 5’-CTGTTGGGGTCTGTCAGGAT |
| Snai2 (Slug) | 5’-CCAAGAAGCCCAACTACAGC | 5’-CGAGGTGAGGATCTCTGGTT |
| T (Brachyury) | 5’-GAGACGGCTGTGGTCCAGTT | 5’-GGGTGGACGAATTCCAGGAT |
| Sox17 | 5’-AACAGCGGGGTGTTCAAATG | 5’-AGCTCTTTTGGCAGGTGTCG |
| Gapdh | 5’-CCCCAACACTGAGCATCTCC | 5’-ATTATGGGGGTCTGGGATGG |
| Goosecoid (Gsc) | 5’-AACGCCGAGAAGTGGAACAA | 5’-AGCTGTCCGAGTCCAAATCG |
| Twist | 5’-CGGAGACCTAGATGTCATTGTT | 5’-CCTCTGGGAATCTCTGTCCA |
| Sox10 | 5’-AGCCCAGGTGAAGACAGAGA | 5’-ATAGGGTCCTGAGGGCTGAT |
